# COVID-19-associated gastrointestinal and liver injury: clinical features and potential mechanisms

**DOI:** 10.1038/s41392-020-00373-7

**Published:** 2020-11-02

**Authors:** Peijie Zhong, Jing Xu, Dong Yang, Yue Shen, Lu Wang, Yun Feng, Chunling Du, Yuanlin Song, Chaomin Wu, Xianglin Hu, Yangbai Sun

**Affiliations:** 1 Department of Musculoskeletal Surgery, Fudan University Shanghai Cancer Center, 200032 Shanghai, China; 2 Department of Oncology, Shanghai Medical College, Fudan University, 200032 Shanghai, China; 3 Department of Gastroenterology and Hepatology, Huaihe Hospital of Henan University, 475000 Kaifeng, China; 4 Clinical Medical College, Southwest Medical University, 646000 Luzhou, China; 5 Department of Endocrinology and Metabolism, Zhongshan Hospital, Fudan University, 200032 Shanghai, China; 6 Department of Pulmonary and Critical Care Medicine, Zhongshan Hospital, Fudan University, 200032 Shanghai, China; 7 Department of Gastroenterology and Hepatology, Zhongshan Hospital, Fudan University, 200032 Shanghai, China; 8 Department of Gastroenterology and Hepatology, Shanghai General Hospital, Shanghai Jiao Tong University School of Medicine, 200080 Shanghai, China; 9 Department of Pulmonary and Critical Care Medicine, QingPu Branch of Zhongshan Hospital Affiliated to Fudan University, 201700 Shanghai, China; 10 Department of Surgery Training Base, Fudan University Shanghai Cancer Center, 200032 Shanghai, China

**Keywords:** Gastrointestinal diseases, Infectious diseases

## Abstract

Coronavirus disease-2019 (COVID-19) is caused by severe acute respiratory syndrome coronavirus 2 (SARS-CoV-2). The infection is spreading globally and poses a huge threat to human health. Besides common respiratory symptoms, some patients with COVID-19 experience gastrointestinal symptoms, such as diarrhea, nausea, vomiting, and loss of appetite. SARS-CoV-2 might infect the gastrointestinal tract through its viral receptor angiotensin-converting enzyme 2 (ACE2) and there is increasing evidence of a possible fecal–oral transmission route. In addition, there exist multiple abnormalities in liver enzymes. COVID-19-related liver injury may be due to drug-induced liver injury, systemic inflammatory reaction, and hypoxia–ischemia reperfusion injury. The direct toxic attack of SARS-CoV-2 on the liver is still questionable. This review highlights the manifestations and potential mechanisms of gastrointestinal and hepatic injuries in COVID-19 to raise awareness of digestive system injury in COVID-19.

## Introduction

Coronavirus disease-2019 (COVID-19) is caused by severe acute respiratory syndrome coronavirus 2 (SARS-CoV-2) and has evolved into a pandemic. Globally, as of 04 October 2020, there have been 34,804,348 confirmed cases of COVID-19, including 1,030,738 deaths, reported by World Health Organization.^1^ Besides common respiratory symptoms, some COVID-19 patients experience gastrointestinal symptoms such as diarrhea, nausea, and vomiting.^2^ Anal swab specimens from COVID-19 patients tested positive for SARS-CoV-2 nucleic acid and SARS-CoV-2 could be isolated from the stool samples of COVID-19 patients,^3,4^ indicating the possibility of fecal–oral transmission. Furthermore, an elevated liver function is common in patients with COVID-19, with more significant increases in alanine aminotransferase (ALT) and aspartate aminotransferase (AST) elevations in severe COVID-19 than in mild/moderate COVID-19.^5^ Thus there is a close relationship between digestive system injury and SARS-CoV-2 infection. This review summarized the manifestations and potential mechanisms of gastrointestinal and hepatic injuries in COVID-19. We aimed to raise awareness of digestive system injury in COVID-19 and provide information for gastrointestinal and hepatic management in COVID-19.

## Mechanism of SARS-CoV-2 infection

**Fig. 1:**
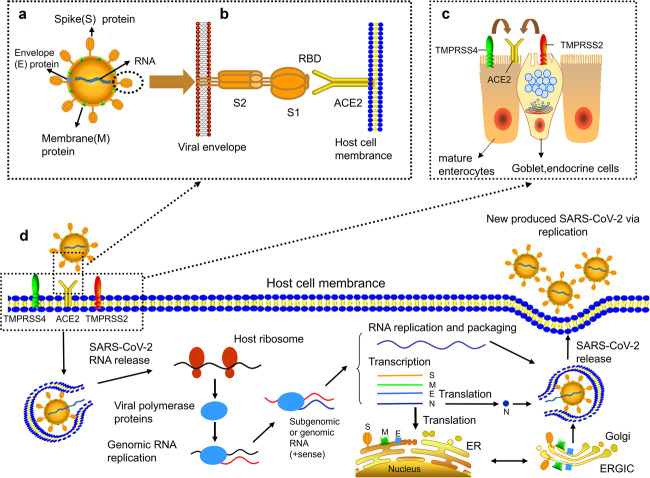
Proposed model of SARS-CoV-2 structure and the life cycle of SARS-CoV-2 in host cells. **a** The structure of SARS-CoV-2. **b** The entry of SARS-CoV-2 into host cells. Transmembrane spike (S) glycoprotein forms homotrimers protruded from the surface of SARS-CoV-2 to recognize human host ACE2 protein. Receptor-binding domain (RBD) is directly involved in the recognition process. **c** TMPRSS2 and TMPRSS4, two mucose-specific serine proteases, can promote the infection of SARS-CoV-2 on ACE2^+^ intestinal epithelial cells. TMPRSS4 is higher expressed than TMPRSS2 in mature enterocytes, while TMPRSS2 is higher expressed than TMPRSS4 in goblet, endocrine cells. **d** Life cycle of SARS-CoV-2 in host cells. First, S protein of SARS-CoV-2 is combined with ACE2 to form ACE2–virus complex. SARS-CoV-2 is transported to host cells with the assistance of TMPRSS2 and TMPRSS4. Second, SARS-CoV-2 RNA is released into host cytoplasm. SARS-CoV-2 RNA conducts translation of viral polymerase proteins via host ribosome. Third, the negative (−)-sense genomic RNA is synthesized and guide synthesis of subgenomic or genomic positive (+)-sense RNA. Nucleocapsids of SARS-CoV-2 are assembled from genomic RNA and N proteins. Other structures of SARS-CoV-2 such as spike (S) protein, envelope (E) protein and membrane (M) protein are translated in the host endoplasmic reticulum (ER). Finally, the viral RNA-N complex and S, M, and E proteins enter ERGIC (endoplasmic reticulum (ER)–Golgi intermediate compartment) and produce a completely new SARS-CoV-2. The new produced SARS-CoV-2 is released from the host cell through exocytosis

The prerequisite for SARS-CoV-2 infection is the entry into host cells, which is dependent on dense glycosylated spike protein (S protein). The S protein contains two functional subunits S1 and S2. The S1 subunit is responsible for binding to host cell receptors, whereas the S2 subunit is responsible for the fusion of viral and cell membranes.^6^ The S protein is initiated by the serine protease TMPRSS2, which is essential for SARS-CoV-2 to enter cells.TMPRSS2 cleaves S protein at S1/S2 and S2 sites.^7^ The S protein of SARS-CoV-2 exists as a trimer, with each monomer containing about 1300 amino acids, of which >300 amino acids constitute the receptor-binding domain (RBD). In particular, the RBD of the S protein domain is directly involved in the recognition of host receptors (Fig. 1).^8–10^ Zhou et al.^11^ demonstrated that the infection of HeLa cells by SARS-CoV-2 depends on the combination between S protein and ACE2. Walls and colleagues^12^ also identified human angiotensin-converting enzyme 2 (ACE2) as a functional receptor for SARS-CoV-2. If the S protein of SARS-CoV-2 is considered key, ACE2 in the human body is like a lock that can be unlocked by S protein. More significantly, based on the results from surface plasma resonance analysis, the S protein of SARS-CoV-2 has 10–20 times the affinity of ACE2 as that of SARS-CoV,^8^ which may explain why SARS-CoV-2 is so contagious.

Other host cell receptors may also mediate SARS-CoV-2 infection. Using genomic receptor profiling with SARS-CoV-2 S protein as the target, Gu et al.^13^ identified 12 surface receptors of SARS-CoV-2, including ACE2. Among them, ASGR1 and KREMEN1 can directly mediate SARS-CoV-2 infection independent of ACE2, hence may be specific receptors of SARS-CoV-2 infection. These multiple host cell receptors of SARS-CoV-2 may explain why SARS-CoV-2 can invade multiple body organs, thus causing complex clinical manifestations. Future studies should detect these potential SARS-CoV-2 receptors other than ACE2 in COVID-19 research as far as possible.

## COVID-19 and gastrointestinal clinical features

Although COVID-19 is mainly a pulmonary disease, gastrointestinal symptoms and signs are prevalent in patients with COVID-19 (Table 1).^4,14–28^ Critically ill patients with COVID-19 had a higher rate of gastrointestinal complications than critically ill patients without COVID-19 (74 vs 37%).^29^ In a cohort study of 1141 confirmed COVID-19 patients, 183 (16%) showed gastrointestinal symptoms. The most common gastrointestinal symptom is lack of appetite, followed by nausea and vomiting.^18^ In a US study of 318 confirmed COVID-19 cases, 61.3% of patients reported at least one gastrointestinal symptom, with loss of appetite (34.8%), diarrhea (33.7%), and nausea (26.4%) being the most common.^23^ Multiple studies from different countries have also reported a variety of gastrointestinal symptoms in COVID-19 patients, particularly diarrhea, nausea, vomiting, and lack of appetite. In a clinical study involving 651 patients with COVID-19, 74 (11.4%) had at least one gastrointestinal symptom (nausea, vomiting, or diarrhea).^30^ A meta-analysis by Parasa et al.^2^ showed that approximately 12% of 4805 patients with COVID-19 presented gastrointestinal symptoms, including diarrhea (4.3–12.2%), nausea, or vomiting (2.6–8.0%). Furthermore, in a group of 204 COVID-19 patients in China, the rate of diarrhea reached to 34.0%.^15^

**Table 1:** Presentation of gastrointestinal symptoms in patients with COVID-19

Reference	Study country	Time of patients’ enrollment	Number of patients	Diarrhea	Nausea	Vomiting	Lack of appetite
Guan et al.^4^	China	December 11, 2019–January 29, 2020	1099	42 (3.8%)	55 (5.0%)^a^	55 (5.0%)^a^	NA
Shi et al.^24^	China	December 20, 2019–January 23, 2020	81	3 (3.7%)	NA	4 (4.9%)	1 (1.2%)
Zhou et al.^19^	China	December 20, 2019–February 9, 2020	254	46 (18.1%)	21 (8.3%)	15 (5.9%)	NA
Luo et al.^18^	China	January 1–February 20, 2020	1141	68 (6.0%)	134 (11.7%)	119 (10.4%)	180 (15.8%)
Wang et al.^14^	China	January 1–28, 2020	138	14 (10.1%)	14 (10.1%)	5 (3.6%)	55 (39.9%)
Zhang et al.^26^	China	January 16–February 3, 2020	139	18 (12.9%)	24 (17.3%)	7 (5.0%)	17 (12.2%)
Mao et al.^22^	China	January 16–February 19, 2020	214	41 (19.2%)	NA	NA	68 (31.8%)
Yang et al.^17^	China	January 17–February 10, 2020	149	11 (7.4%)	2 (1.3%)	2 (1.3%)	NA
Lin et al.^20^	China	January 17–February 15, 2020	95	23 (24.2%)	17 (17.9%)	4 (4.2%)	17 (17.9%)
Pan et al.^15^	China	January 18–February 28, 2020	204	35 (34.0%)	NA	4 (3.9%)	81 (78.6%)
Wan et al.^16^	China	January 19–March 6, 2020	232	49 (21.1%)	NA	NA	NA
Lu et al.^25^	China	January 28–February 26, 2020	171	15 (8.8%)	NA	11 (6.4%)	NA
Zheng et al.^21^	China	February 5–March 9, 2020	1320	107 (8.1%)	57 (4.3%)	57(4.3%)	62 (4.7%)
Argenziano et al.^28^	USA	March 1–April 5, 2020	1000	236 (23.6%)	178 (17.8%)^b^	178 (17.8%)^b^	NA
Suleyman et al.^27^	USA	March 9–March 27, 2020	463	100 (21.7%)	94 (20.4%)	53 (11.5%)	100 (21.7%)
Redd et al.^23^	USA	Before April 2, 2020	318	107 (33.7%)	84 (26.4%)	49 (15.4%)	110 (34.8%)

*NA* not available

^a^The number of cases with nausea or vomiting is 55 (5.0%)

^b^The number of cases with nausea or vomiting is 178 (17.8%)

It is worth noting that gastrointestinal symptoms such as diarrhea may appear in some cases earlier than fever and respiratory symptoms. In a family cluster of six patients, two had diarrhea as an initial symptom and were admitted to hospital without fever.^31^ In a Chinese cohort of 138 COVID-19 patients, 14 (10.1%) patients had diarrhea and nausea symptoms for 1–2 days before reporting fever and dyspnea.^14^ The first COVID-19 case in the US had a history of nausea and vomiting for 2 days before admission, with diarrhea being reported the next day.^3^ In a US cohort, patients with gastrointestinal symptoms (defined as diarrhea or nausea/vomiting) were more likely to test positive for COVID-19 than those without gastrointestinal symptoms (61 vs 39%).^32^ Therefore, special attention should be paid to patients with gastrointestinal symptoms during the COVID-19 pandemic.

Compared with patients without gastrointestinal symptoms, patients with gastrointestinal symptoms take a long time from COVID-19 onset to admission (9.0 vs 7.3 days).^15^ As the epidemic progressed, the rate of diarrhea reported in hospitalized COVID-19 patients seemed to be increasing.^16^ Presence of diarrhea is correlated with the severity of COVID-19. Indeed, more critically ill patients have diarrhea.^16^ Besides, Cholankeril et al.^33^ found that the incidence of acute renal insufficiency is higher in COVID-19 patients with gastrointestinal symptoms than those without gastrointestinal symptoms (9.3 vs 3.1%). COVID-19 patients hospitalized on medical floors and in intensive care units (ICU) had a higher prevalence of gastrointestinal symptoms than patients observed only in the emergency room (60.0 vs 23.5%). Hoel et al.^34^ assessed marker of intestinal epithelial cell damage (intestinal fatty acid-binding protein), marker of intestinal leakage (lipopolysaccharide-binding protein (LBP)), marker of intestinal homing (C-C chemokine motif ligand 25 (CCL25)), and markers of inflammasome activation (interleukin (IL)-1, IL-18) in plasma between 39 COVID-19 patients and 16 healthy controls. Compared with the controls, LBP and CCL25 were significantly increased in COVID-19 patients. Plasma LBP and inflammasome activation markers were significantly increased in COVID-19 patients with cardiac involvement. Impaired intestinal functional barriers and increased inflammasome activation may promote cardiac involvement in COVID-19 patients. Wan et al.^16^ also reported that COVID-19 patients with diarrhea required more ventilator support and intensive care than those without diarrhea. However, a short-term follow-up cohort by Nobel et al.^32^ showed that mortality is lower in COVID-19 patients with gastrointestinal symptoms compared to those without symptoms (0.0 vs 5.0%), with no statistical significance in the ICU admission rate between COVID-19 patients with and without gastrointestinal symptoms. More clinical data are required to further explore the relationship between COVID-19 severity and the symptoms of gastrointestinal injury.

## Mechanism of SARS-CoV-2 infection of the gastrointestinal tract

Bioinformatics analysis based on single-cell transcriptome showed that ACE2 is not only highly expressed in the lung AT2 cells but also in the esophagus upper and stratified epithelial cells and absorptive enterocytes from the ileum and colon.^35^ In human small intestinal organoids, enterocytes can be infected by SARS-CoV and SARS-CoV-2.^36^ Zang et al.^37^ concluded that the expression of ACE2 is significantly higher in human and mouse small intestine than in all other organs, including lungs. In addition, they used a chimeric vesicular stomatitis virus green fluorescent protein reporter virus in which the native glycoprotein (G) is genetically replaced with SARS-CoV-2 S protein. They confirmed that SARS-CoV-2 could infect human intestinal enteroids and replicate in ACE2^+^ mature enterocytes. Furthermore, TMPRSS2 and TMPRSS4, two transmembrane protease serines, can promote SARS-CoV-2 infection of human small intestinal enterocytes. Nasopharyngeal aspirates obtained from three COVID-19 patients were co-cultured with human or bat intestinal organs, with the intestinal organs showing an obvious cytopathic response and a rapid increase in the SARS-CoV-2 load. The transcription and expression levels of ACE2 and TMPRSS2 (the requirements for SARS-CoV-2 invasion into host cells) significantly increased in the induced differentiation of human intestinal organs. Crucially, both bat and human intestinal organs maintained SARS-CoV-2 replication, with intestinal cells being the primary target of SARS-CoV-2.^38^ Currently, the exact mechanism of SARS-CoV-2 interaction with the gastrointestinal tract is still not fully understood. However, according to current evidence, it remains a key question that the gastrointestinal symptoms in COVID-19 are somehow caused by the direct attack of SARS-CoV-2 to gastrointestinal tract.

## COVID-19 and the fecal–oral transmission route

It has been shown that SARS-CoV inoculated into hospital sewage remains infectious for 2 weeks.^39^ Middle East respiratory syndrome (MERS)-CoV, which is also a coronavirus, can survive for 48 h in a low temperature and humid environment and for 8 h at 30 °C and 80% phase humidity bar.^40^ The researchers detected positive nucleic acids of MERS-CoV in the stool samples of patients with MERS. After the inoculation of MERS-CoV directly into the stomach of hDPP4 transgenic mice, MERS-CoV could not only survive and replicate in the intestines of mice but also develop intestinal inflammation in infected mice.^41^ Although at present the biological characteristics and temperature sensitivity of SARS-CoV-2 are not clear, since both SARS-CoV and MERS-CoV have evidence of fecal excretion, a fecal–oral transmission route of SARS-CoV-2 is possible. Coincidentally, a stool sample of the first confirmed COVID-19 patient in the US tested positive for SARS-CoV-2 nucleic acid.^3^ Guan et al.^4^ analyzed 62 stool samples in their study on the clinical characteristics of SARS-CoV-2 infection in China, finding four (6.5%) samples were positive for SARS-CoV-2. These findings provide evidence for gastrointestinal infection of SARS-CoV-2 and support that fecal–oral transmission may be a potential route of transmission. Unlike SARS in 2003, which normally took 2–3 weeks to peak in the intestinal tract, a study found that SARS-CoV-2 had the highest positive rate of elimination (44.19%) in the early stage of infection (the first week of the course). In the progression of SARS-CoV-2 infection, the positive rate of SARS-CoV-2 virus nucleic acid detection in feces gradually decreases, forming a plateau in the course of 4–5 weeks.^42^

The viral load measured from tissue samples indicates that SARS-CoV-2 is actively replicating.^43^ In a cohort study of 59 COVID-19 patients in Hong Kong, 15 (25.4%) had gastrointestinal symptoms, while 9 (15.3%) had a viral RNA positive stool. The median viral load in feces of patients with diarrhea was higher than that of patients without diarrhea (5.1log_10_ copies per milliliter vs 3.9log_10_ copies per milliliter).^44^ Zheng et al.^43^ collected 3497 respiratory, stool, serum, and urine samples from 96 confirmed COVID-19 patients after admission and detected SARS-CoV-2 in stool samples from 55 patients (59%). Compared with respiratory samples, the duration of the virus was longer and the peak time of viral load was later. Although there was no significant difference in viral loads in stools between patients with mild COVID-19 and those with severe COVID-19, the viral load was highest during the third and fourth weeks after disease onset.

It is a key question that long isolation and detection of SARS-CoV-2 is required in excrement and urine. In a study of 73 hospitalized patients with SARS-CoV-2 infection, 39 (53.42%) were positive for SARS-CoV-2 RNA in stools, and 17 (23.29%) were positive for SARS-CoV-2 RNA in stools even after the respiratory virus was negative.^45^ In another clinical cohort of 74 patients, 41 (55%) tested positive for SARS-CoV-2 RNA in fecal and respiratory samples.^46^ The average duration of positive respiratory samples is 16.7 days and 27.9 days for positive stool samples, which is 11.2 days longer than that of respiratory samples.^46^ More seriously, in a complete disease course of 41 patients with positive stool samples, 1 patient remained positive for 33 consecutive days after the respiratory sample turned negative, while another remained positive for 47 days after the onset of illness.^46^

Furthermore, Xiao et al.^47^ isolated SARS-CoV-2 from the stools of a deceased COVID-19 patient, suggesting fecal–oral or fecal–respiratory transmission, which warrants further study. Jeong et al.^48^ failed to directly prove the presence of viable SARS-CoV-2 in stool samples by cell culture isolation. However, they were able to isolate SARS-CoV-2 from animals (two of two ferrets) when they inoculated infantile ferrets with a fecal sample from a COVID-19 patient. Although the infectious viral load for a ferret might be different from humans, the presence of infectious SARS-CoV-2 in stool samples deserves special attention.

Therefore, we recommend strict disinfection in the treatment of feces and other sewage from patients with COVID-19. Fecal–oral transmission should be considered to minimize the risk of nosocomial infection.

## COVID-19 and liver-associated clinical features

**Fig. 2:**
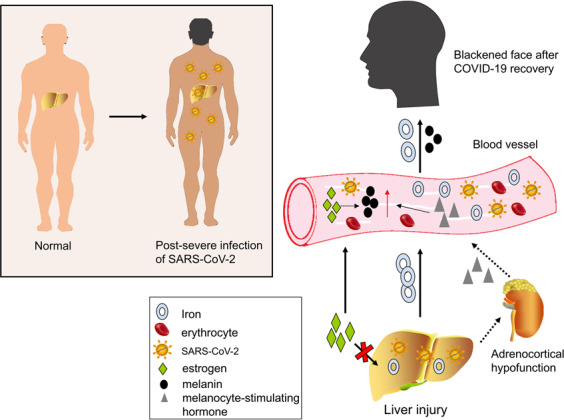
Patients with severe COVID-19 develop facial blackness and dull skin after recovery. Liver injury during COVID-19 is mainly responsible for these special manifestations. Three possible mechanisms are presented: (1) iron in the damaged liver drains into blood vessels. The blood with high iron level can lead to a blackening of the face once it supplies to the facial skin; (2) estrogen cannot be metabolized in the damaged liver. An increase in estrogen in the blood eventually causes an increase in conversion of tyrosine to melanin; (3) when liver function is impaired, adrenocortical function is hypoactive and melanocyte-stimulating hormone increases

In China, it was reported that some patients who recovered from severe COVID-19 suffered special manifestations of a darkened face and pigmentation during recovery. Multiple organ injury, especially liver injury, is mainly responsible for the darkened face and pigmentation.^49,50^ Abnormal liver function can easily lead to pigmentation via different three pathways: (1) liver dysfunction can hinder inactivation of estrogen.^51^ The increase of estrogen reduces the inhibition of thiamine on tyrosinase in vivo, thus increasing the conversion of tyrosine into melanin;^52^ (2) abnormal liver function can lead to adrenocortical hypofunction. The liver cannot metabolize the melanocyte-stimulating hormone secreted by the anterior pituitary gland, which causes increased secretion of melanin;^53,54^ (3) a liver injury can increase the iron level in the blood, which if supplied to the facial skin, can cause a blackened face^55,56^ (Fig. 2).

In addition to gastrointestinal symptoms, liver enzyme levels are reportedly elevated in 7.6–39% of COVID-19 patients. In a US cohort study of 5700 COVID-19 patients, abnormalities in ALT were observed in 2176 (39.0%) patients and AST in 3263 (58.4%) patients.^57^ Coincidentally, patients infected with SARS-CoV and MERS-CoV also have liver enzyme abnormalities (Table 2). When patients are infected with SARS-CoV-2, typically they have mildly to moderately elevated ALT and/or AST in the early stages of the disease, accompanied by a slight increase in bilirubin levels.^4,5,17,57–70^ However, increased ALT and AST levels of 7590 and 1445 U/L, respectively, have been reported in a few patients.^59^ Studies have shown that the prevalence of elevated transaminase and bilirubin is at least twice as high in severe patients than in mild and moderate patients.^71^

**Table 2:** Abnormal liver enzyme levels in peripheral blood: a comparison of COVID-19, SARS, and MERS

Reference	Study country	Number of patients	Elevated serum level		
			Abnormal ALT, no. (%)	Abnormal AST, no. (%)	Abnormal TBil, no. (%)
*COVID-19*					
Wang et al.^5^	China	105	40 (38.1%)	33 (31.4%)	24 (22.9%)
Guan et al.^4^	China	1099	158 (21.3%)^a^	168 (22.2%)^b^	76 (10.5%)^c^
Yang et al.^17^	China	149	18 (12.1%)	27 (18.1%)	4 (2.7%)
Fan et al.^58^	China	148	27 (18.2%)	32 (21.6%)	9 (6.1%)
Chen et al.^59^	China	99	28 (28.3%)	35 (35.4%)	18 (18.2%)
Zhang et al.^60^	China	82	22 (30.6%)^d^	44 (61.1%)^d^	22 (30.6%)^d^
Huang et al.^61^	China	36	4 (13%)	18 (58.1%)	4 (12.9%)
Richardson et al.^57^	USA	5700	2176 (39.0%)	3263 (58.4%)	NA
Wu et al.^62^	China	157	12 (7.6%)	25 (16.9%)	NA
*SARS*					
Cui et al.^63^	China	182	128 (70.3%)	57 (31.3%)	NA
Duan et al.^64^	China	154	41 (26.6%)	4 (2.6%)	13 (8.4%)
Zhang et al.^65^	China	128	54 (42.2%)	NA	17 (13.3%)
*MERS*					
Yousefi et al.^66^	Iran	5	2 (40%)	3 (60%)	NA
Assiri et al.^67^	Saudi Arabia	47	5 (11.0%)	7 (15.0%)	NA
Al-Tawfiq et al.^68^	Saudi Arabia	17	3 (17.6%)	9 (52.9%)	NA
Arabi et al.^69^	Saudi Arabia	330	142/252 (56.3%)^e^	197/227 (86.8%)^f^	NA
Al Ghamdi et al.^70^	Saudi Arabia	51	23 (45.1%)	35 (68.6%)	NA

*NA* not available

^a^Includes data for 741 patients

^b^Includes data for 757 patients

^c^Includes data for 722 patients

^d^Includes data for 72 patients

^e^Includes data for 252 patients

^f^Includes data for 227 patients

## Mechanism of COVID-19-associated liver injury

Drug-induced liver injury in COVID-19 is possible. The main manifestation of COVID-19, fever, leads to the usage of antipyretic drugs, which contain acetaminophen, a drug that commonly causes liver injury.^72^ Other hepatotoxic drugs, lopinavir/ritonavir, oseltamivir, interferon, antibacterial agents, and Chinese herb have been widely used in China to fight COVID-19. Zhan et al.^73^ found that the use of lopinavir/ritonavir is an independent risk factor for severe liver damage in COVID-19 patients. For patients with hypohepatia, those hepatotoxic drugs should be used with caution. Combination of multiple hepatotoxic drugs must be avoided. Liver function should be monitored during administration.

Moreover, COVID-19-induced liver injury may be closely related to systemic inflammatory response syndrome (SIRS). In some COVID-19 patients, the disease is not serious in the early stage but they suddenly deteriorate quickly entering a state of multi-organ failure.^74,75^ The inflammatory cytokines storm induced by an excessive immune response may be the culprit. A cohort study of 192 COVID-19 patients showed that increased IL-6 and IL-10 and decreased CD4^+^ T cells were independent risk factors for severe liver damage.^73^ High levels of IL-1β, IL-2, IL-6, IL-8, IL-10, IL-17, interferon, IP10, and monocyte chemoattractant protein 1 in patients infected with SARS-CoV-2 have also been observed.^74,76^ Much release of inflammatory cytokines by immune cells triggers acute respiratory distress syndrome (ARDS) and SIRS. Such a vicious systematic inflammatory cytokines storm not only leads to lung injury but also liver injury. Furthermore, inflammasome activation has been found to participate in COVID-19. The activation of inflammasome-related cytokines is related to the COVID-19 severity. Inflammasome activation may link to the poor prognosis and death of some COVID-19 patients.^77^ Inflammasome activation and apoptosis/pyroptosis in SARS-CoV-2-induced alveolar macrophages and recruited monocyte-derived macrophages may significantly exacerbate COVID-19 symptoms.^78^

Current opinion considers COVID-19 indeed is a kind of vascular disease, with coagulopathy and thrombosis. SARS-CoV-2 may infect endothelial cells and cause diffuse endotheliitis. Subsequent microvascular dysfunction leads to hypercoagulability, tissue edema, and organ ischemia.^79,80^ Hepatic ischemia–reperfusion injury (HIRI) is a common pathophysiological process. The main mechanism is closely related to reactive oxygen species, neutrophils, Kupffer cells, and calcium overload. Hepatic ischemia–reperfusion can activate Kupffer cells, neutrophils, and platelets, causing a series of destructive cellular reactions, leading to inflammation and cell injury. Meanwhile, microcirculation disorder caused by the injury of hepatic sinusoidal endothelial cells can further aggravate liver ischemia and oxygen deficiency. More than 40% of COVID-19 patients with various degrees of hypoxemia require oxygen therapy.^4^ It is generally accepted that liver injury often occurs in patients with hypotensive shock or severe hypoxemia. The hypoxic internal environment caused by severe COVID-19 can lead to ischemia hypoxia reperfusion liver injury. Moreover, lymphatic vessels are involved in the pathological process of acute liver injury (ALI)/ARDS and play an important role in preventing the occurrence of ALI/ARDS and delaying the progression of COVID-19.^81^ Lymphatic vessels are involved in virus clearance by absorbing and transporting large amounts of exudate produced by inflammation, inflammatory cytokines, and dead cell debris and transporting immune cells, such as T cells.^82^

The mechanism of liver injury during SARS-CoV-2 infection is still under investigation. The world’s first pathological autopsy of COVID-19 revealed that the liver tissue shows moderate microvesicular steatosis and mild lobular activity.^75^ Liver injury in COVID-19 may be due to the direct invasion of SARS-CoV-2 and the destruction of hepatocytes. ACE2 is specifically expressed in the bile duct epithelium and is 20 times higher than in hepatocytes.^83^ It is suggested that compensatory proliferation of liver parenchymal cells derived from bile duct cells leads to the upregulation of the overall expression of ACE2 in liver tissue, which may be one of the mechanisms of liver injury caused by SARS-CoV-2 infection. Wang et al.^84^ proposed that SARS-CoV-2 could directly contribute to cytopathy based on the ultrastructural findings of conspicuous mitochondria swelling, endoplasmic reticulum dilatation, glycogen granule decrease, and impaired cell membranes. Histologically, numerous apoptotic hepatocytes and some binuclear hepatocytes were observed. However, the conclusion of Wang et al.^84^ was supported only by simple tissue pathology and single transmission electron microscopic imaging of part of a cell claimed to be hepatocyte; statistics was not even available. Therefore, it must be interpreted with caution that SARS-CoV-2 might infect the liver and is a key factor in liver dysfunction. The direct toxic attack of SARS-CoV-2 on the liver is still questionable and needs more evidences.

**Fig. 3:**
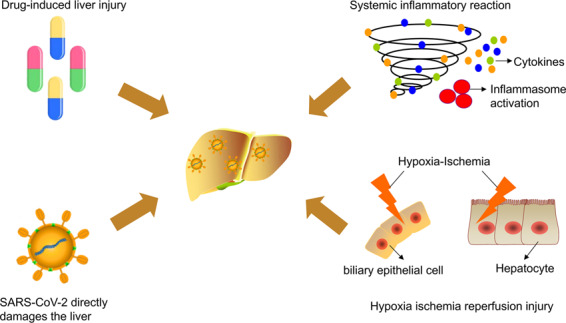
Mechanisms of COVID-19-associated liver injury: (1) drug-induced liver injury; (2) systemic inflammatory response (inflammatory cytokine storm); (3) hypoxic ischemia–reperfusion injury; (4) direct toxic effect of SARS-CoV-2 on the liver

Indeed, current evidence supports that COVID-19-associated liver injury is a multifactorial attack including drug-induced liver injury, systemic inflammatory reaction, hypoxia ischemia reperfusion liver injury, and possible direct injury by SARS-CoV-2 to the liver (Fig. 3).

## COVID-19 in liver transplant patients

The global spread of COVID-19 poses new challenges to organ donation and transplantation. Transplant recipients with preoperative organ decompensation and chronic disease are more likely to contract respiratory viruses. Liver transplant recipients who are exposed to more people while waiting for transplantation have high risk factors for cross-infection and COVID-19 epidemiology. Qin et al.^85^ reported a case of COVID-19 infection after liver transplantation for hepatocellular carcinoma. The patient’s thorax computed tomography showed multicentric subpleural ground glass opacification in the left lobes on the 11th day after liver transplantation, and the following day, the patient was diagnosed with COVID-19 after a positive nucleic acid test. Liver transplantation recipients take long-term immunosuppressants to prevent rejection reaction, which may significantly reduce their ability to defend against COVID-19 infection due to a weakened immune system. A systematic review of 223 liver transplant patients with confirmed COVID-19 from 15 studies showed that liver transplant patients were more likely to present with concurrent diarrhea. Elderly COVID-19 patients with dyspnea and diabetes had a higher mortality rate of nearly 23%.^86^ However, current data have not yet supported that liver transplant recipients are susceptible to COVID-19. A series of cases in Italy reported that children who receive liver transplants, despite being immunosuppressed, have no increased risk of developing severe lung disease compared to the general population.^87^ Similarly, all three COVID-19-related deaths observed by Bhoori et al.^88^ at an Italian transplant center were long-term patients on a minimal immunosuppression regimen, rather than recently transplanted patients with complete immunosuppression. Webb et al.^89^ conducted a large international observational study and showed that liver transplantation cannot significantly increase the risk of death in COVID-19 patients.

## COVID-19 and proton pump inhibitors (PPIs)

PPIs are commonly used to treat patients with gastroesophageal reflux disease and peptic ulcer disease. PPI is the most effective gastric acid secretion inhibitor, reducing the acid released into the stomach by blocking the proton pump. While the reduction of stomach acid may be beneficial to patients with stomach disorders, it may leave the gut vulnerable to coronavirus infection.^90,91^ Xiao et al.^92^ suggested that the S protein of SARS-CoV mediated the fusion with host cells under neutral pH conditions. Darnell et al.^93^ confirmed that extremely alkaline pH 12 and 14 and highly acidic pH 1 and 3 can lead to the inactivation of SARS-CoV, while the virus can remain stable within a range of neutral pH. Similarly, SARS-CoV-2 is still alive on the sixth day but lose between 2.9 and 5.33 logs of infectivity at pH 5–9. At pH extremes (pH 2–3 and pH 11–12), SARS-CoV-2 lost infectivity within 1 day.^94^ Zhou et al.^95^ reported that SARS-CoV-2 was completely inactivated and incapable of infecting cells under the conditions of pH 1.0 and 2.0 (similar to the normal acidity of an empty stomach) by making viruses pseudotyped with SARS-CoV-2 S protein. Stomach secretions have a pH between 1.0 and 3.5, while the small and large intestine have a pH between 7.5 and 8.0. Ingestion of SARS-CoV-2 may cause most virus particles to be inactivated by gastric acid. However, if a person takes an acid suppressant such as PPI for a long period of time, their stomach acidity will decrease. So, SARS-CoV-2 may have an increased chance to enter the gut from the stomach and lead to viral infection.

Current clinical data well supported that PPIs facilitate COVID-19 infection and aggravate COVID-19. Almario et al.^96^ conducted a cohort study of 53,130 individuals, of whom 3386 (6.4%) were infected with COVID-19. When compared to individuals not using PPIs, those taking PPIs up to once or twice daily had significantly increased odds of a positive COVID-19 test. However, patients taking histamine-2 receptor antagonists were not at an elevated risk. In addition, patients who take just two doses of PPIs a day are more likely to be positive for COVID-19 than those who take just one dose. COVID-19 patients taking lower-dose PPIs have lower odds for gastrointestinal symptoms vs those not taking PPIs. In a Korean cohort of 111,911 unused PPIs users, 14,163 current PPI users, and 6242 past PPI users, Lee et al.^97^ found that, although PPIs usage did not increase COVID-19 infection, the current use of PPIs was associated with a 79% increased risk of severe clinical outcomes of COVID-19. PPIs use in the past 30 days increased the risk of severe clinical outcome for COVID-19 by 90%. Thus PPI medication should be paid special attention in COVID-19.

Furthermore, there are also many other drugs commonly used in patients with gastrointestinal diseases deserving to be elucidated in COVID-19 in the future. For example, for patients with inflammatory bowel disease (IBD), it is interesting to explore whether antitumor necrosis factor drugs, vedolizumab, ustekinumab, and steroids affect COVID-19 infection and severity. Kumar et al.^98^ considered that immunosuppressive drug therapy may increase the risk of COVID-19 infection in IBD patients. IBD patients should be paid special attention to COVID-19 vaccination.

## In summary

In this review, we summarized recent reports of gastrointestinal symptoms and liver biochemical abnormalities in COVID-19 patients until September 2020. Gastrointestinal and liver dysfunction as a sign of early SARS-CoV-2 infection should not be neglected during the COVID-19 pandemic. Considering that stools can be positive for SARS-CoV-2 nucleic acids even when respiratory samples are negative, the preventive management of fecal–oral transmission deserves close attention. Timely correction of primary disease (SARS-CoV-2 infection) remains the most key to cure of COVID-19-associated gastrointestinal and liver injury. Moreover, intestinal microecological regulators are suggested to maintain intestinal microecological balance and prevent secondary bacterial infection via gut barrier. Hepatic protectants can also be appropriately considered in severe COVID-19 patients. The mechanisms of COVID-19-associated gastrointestinal and liver injury are extremely multidimensional, hence further translational and basic research is urgently needed to reveal the intrinsic relationship of COVID-19 with gastroenterology and hepatology.
